# Preoperative aspartate transaminase/alanine transaminase ratio as a prognostic biomarker in primary non-muscle-invasive bladder cancer: a propensity score-matched study

**DOI:** 10.1186/s12894-021-00901-9

**Published:** 2021-09-27

**Authors:** Xiaofeng Cheng, Xiaochen Zhou, Ming Yi, Song Xu, Cheng Zhang, Gongxian Wang

**Affiliations:** Department of Urology, The First Affiliated Hospital of Nanchang University, Nanchang City, 330000 Jiangxi Province China

**Keywords:** Aspartate transaminase, Alanine transaminase, Primary non-muscle-invasive bladder cancer, Survival prognosis

## Abstract

**Purpose:**

To evaluate the prognostic value of the aspartate transaminase/alanine transaminase (AST/ALT) ratio in primary non-muscle-invasive bladder cancer (NMIBC) using propensity score matching (PSM) analysis.

**Methods:**

We retrospectively collected the clinical and pathological data from 314 patients with primary NMIBC who underwent transurethral resection of bladder tumor. The full cohorts were divided into a low AST/ALT ratio group and a high AST/ALT ratio group according to the optimal cut-off value which was obtained based on the analysis of the receiver operating characteristic curve for the 3-year recurrence-free survival (RFS). After 1:1 PSM, the correlation between preoperative AST/ALT ratio and survival prognosis was evaluated by Kaplan–Meier analysis with log-rank tests. The independent prognostic factors for RFS and progression-free survival (PFS) were also analyzed.

**Results:**

The optimum cutoff value of the preoperative AST/ALT ratio was 1.40. Before PSM, a high AST/ALT ratio was correlated with the larger proportion of age > 60 years (*P* = 0.007) and the worse pathological T stage (*P* < 0.001). After PSM, patients with a high AST/ALT ratio had poorer RFS and PFS than patients with a low AST/ALT ratio (all *P* < 0.001). In addition, multivariate Cox regression analysis indicated that preoperative AST/ALT ratio was considered as an independent prognostic factor of RFS (HR 2.865; 95%CI 1.873–4.381; *P* < 0.001) and PFS (HR 4.771; 95%CI 2.607–8.734; *P* < 0.001) in patients with primary NMIBC.

**Conclusions:**

The high AST/ALT ratio group tended to have poorer RFS and PFS than the low AST/ALT ratio group. Our results also indicated that the elevated preoperative AST/ALT ratio could be seen as a useful prognostic biomarker for predicting early disease recurrence and progression in patients with primary NMIBC.

## Background

Bladder cancer is one of the most prevalent cancers in the urinary tract and the seventh most common tumor in the male population worldwide, while it drops to the 11th if both genders are considered [1, 2]. Approximately 75% of patients with bladder cancer present with disease confined to the mucosa or submucosa, which is referred to as non-muscle-invasive bladder cancer (NMIBC) and this proportion is even higher in younger patients (< 40 years) [3]. The standard treatment for NMIBC is transurethral resection of bladder tumor (TURBT), eventually followed by adjuvant intravesical therapy based on risk stratification [4]. However, the high recurrence rate of NMIBC patients is as high as 50–70% and one-third of recurrent patients will develop into muscle-invasive bladder cancer (MIBC) and eventually succumb to the disease [5, 6]. To better predict the recurrence and progression of NMIBC, several biomarkers, such as serum cholinesterase, neutrophil-to-lymphocyte ratio, plasma fibrinogen, and D-dimer, have been studied [7–9]. Nevertheless, our knowledge of plasma prognostic factors assessed preoperatively in NMIBC remains limited.

Aspartate transaminase (AST) and alanine transaminase (ALT) are found in the liver, heart, skeletal muscle, brain, kidney, and red blood cells, which are widely used to reflect hepatocellular damage in clinical practice. In 1957, the ratio of the serum activities of AST to ALT was first described as an important indicator for differentiating the etiology of acute hepatitis by De Ritis et al., which was also called the De Ritis ratio [10]. The AST/ALT ratio was widely used to identify various liver diseases such as viral hepatitis and alcoholic hepatitis. Meanwhile, the latest research shows that this ratio has been reported as a prognostic biomarker in several malignancies, such as renal cell carcinoma, lung cancer, pancreatic cancer, and colorectal cancer [11–15]. Similarly, this ratio has been demonstrated as a significant prognostic biomarker in patients undergoing radical cystectomy with bladder cancer [16–18]. Although their study was valuable for proposing the AST/ALT ratio as a new prognostic factor in BC, the molecular features and clinical outcomes between NMIBC and MIBC are highly distinct [19]. The prognostic predictive value of the AST/ALT ratio in primary NMIBC needs to be further clarified.

The purpose of this study was to evaluate the prognostic predictive value of the AST/ALT ratio on the survival prognosis of patients with primary NMIBC. A propensity score-matching (PSM) methodology was performed to minimize the interference of other confounding factors.

## Methods

### Patient selection

We retrospectively reviewed the relevant medical records and collected clinical and pathological data of 314 patients with primary NMIBC who underwent TURBT at the Department of Urology of the First Affiliated Hospital of Nanchang University from January 2013 to May 2017. Patient inclusion criteria as following: (1) biopsy of cystoscopy and postoperative pathological outcomes were consistent in confirming non-muscle-invasive bladder urothelial carcinoma, (2) laboratory data of AST and ALT were obtained within 5 days before the first surgical treatment, and (3) adequate follow-up information is available for prognostic analysis. Exclusion Criteria: (1) any concomitant malignancy and pelvic radiation, (2) present hematological disorders and chronic liver disease within the last 3 months, (3) perioperative death. All patients obtained and signed informed consent. This study has been approved by the Institutional Ethical Review Board and the study protocol was carried out following the guidelines of the Helsinki declaration.

### Study design

To evaluate the optimum thresholds for the AST/ALT ratio, the receiver operating characteristic curve for 3-year recurrence-free survival (RFS) was analyzed. The optimum threshold for the AST/ALT ratio was obtained when there was a maximum Youden index value. This threshold transforms the AST/ALT rate into a dichotomous variable that divides all patients into a high AST/ALT group and a low AST/ALT group. To balance the differences of the covariates of baseline data in both the group, PSM analysis was done using a multivariable logistic regression model based on age, gender, the history of smoking, tumor size, tumor number, restaging transurethral resection of the bladder tumor (Re-TURBT), pathological stage, pathological grade, concomitant carcinoma in situ, type of intravesical therapy. Pairs of patients were derived using 1:1 greedy nearest neighbor matching within a caliper size of 0.1. Further, the clinicopathological data of the two paired groups were analyzed and the predictive value of the AST/ALT ratio in primary NMIBC was evaluated.

### Patient management and follow‑up

TURBT was performed by skilled urologists at our center. Indications for Re-TURBT in patients with NMIBC remain controversial [20]. Thus, Re-TURBT was not routinely performed in all patients. All patients received postoperative observation, early single instillation, adjuvant intravesical chemotherapy, or intravesical BCG (Bacillus Calmette-Guerin) treatment according to tumor characteristics. All pathological diagnoses were made by two pathologists after viewing and checking. In this study, the International Union Against Cancer tumor, node, metastasis classification system (2009) and the World Health Organization International Society of Urologic Pathology (2004) was used as the standard for bladder cancer staging and grading. The patient follow-up protocol which included surveillance cystoscopy or computerized tomography of the pelvis was performed at 3-month intervals for the initial 2 years, every 6 months in the next 3 years, and annually thereafter. RFS was defined as the time interval from initial TURBT to the first occurrence of local or distant recurrent disease. Progression-free survival (PFS) referred to the interval from initial surgery to first disease progression (any advance in tumor grade or stage).

### Statistical analysis

All categorical data were presented as a percentage or an absolute number, while continuous variables were presented as the median and interquartile range (IQR). The difference in clinicopathologic factors between the two groups was taken by the chi-square test or Fisher’s exact test before PSM, while the McNemar test was performed after PSM. The difference in survival was evaluated by Kaplan–Meier analysis with log-rank tests. The univariate and multivariate analyses were performed by a Cox proportional hazard model to identify the potential predictors of prognosis. PSM was performed using the MatchIt package in R software; all statistical analyses were performed using SPSS 26.0 Statistics software. *P* < 0.05 was considered to be statistically significant.

## Results

### Clinical and pathological characteristics in the full cohorts

A total of 314 patients newly diagnosed with NMIBC who underwent TURBT and were included in this study. The study population included 250 male and 64 female patients with a median age of 65 years (IQR 58–73 years), 137of whom had a history of smoking. 86 patients had a history of Re-TURBT. The tumor size was ≤ 3 cm in 247 (78.7%) patients, and > 3 cm in 67 (21.3%) patients. Multifocal tumors were found in 164 (52.2%) patients. Pathological T stage was Ta in 161 (51.3%) and T1 in 153 (48.7%) patients. Tumor grade was papillary urothelial neoplasms of low malignant potential in 13 (4.1%), low grade in157 (50.0%), and high grade in 144 (45.9%) patients. 29 (9.2%) patients had concomitant carcinoma in situ (CIS). 34 (10.8%) patients underwent early single instillation only and 48 (15.3%) patients were treated with postoperative adjuvant chemotherapy or BCG only, while 232 (73.9%) patients underwent combined therapy. Furthermore, 171 (54.5%) patients experienced recurrence, and 94 (29.9%) patients experienced progression after initial TURBT. The median follow-up time was 48 months (IQR 22.3–72 months).

### Determination of optimal cutoff values regarding prediction of 3-year RFS

**Fig. 1:**
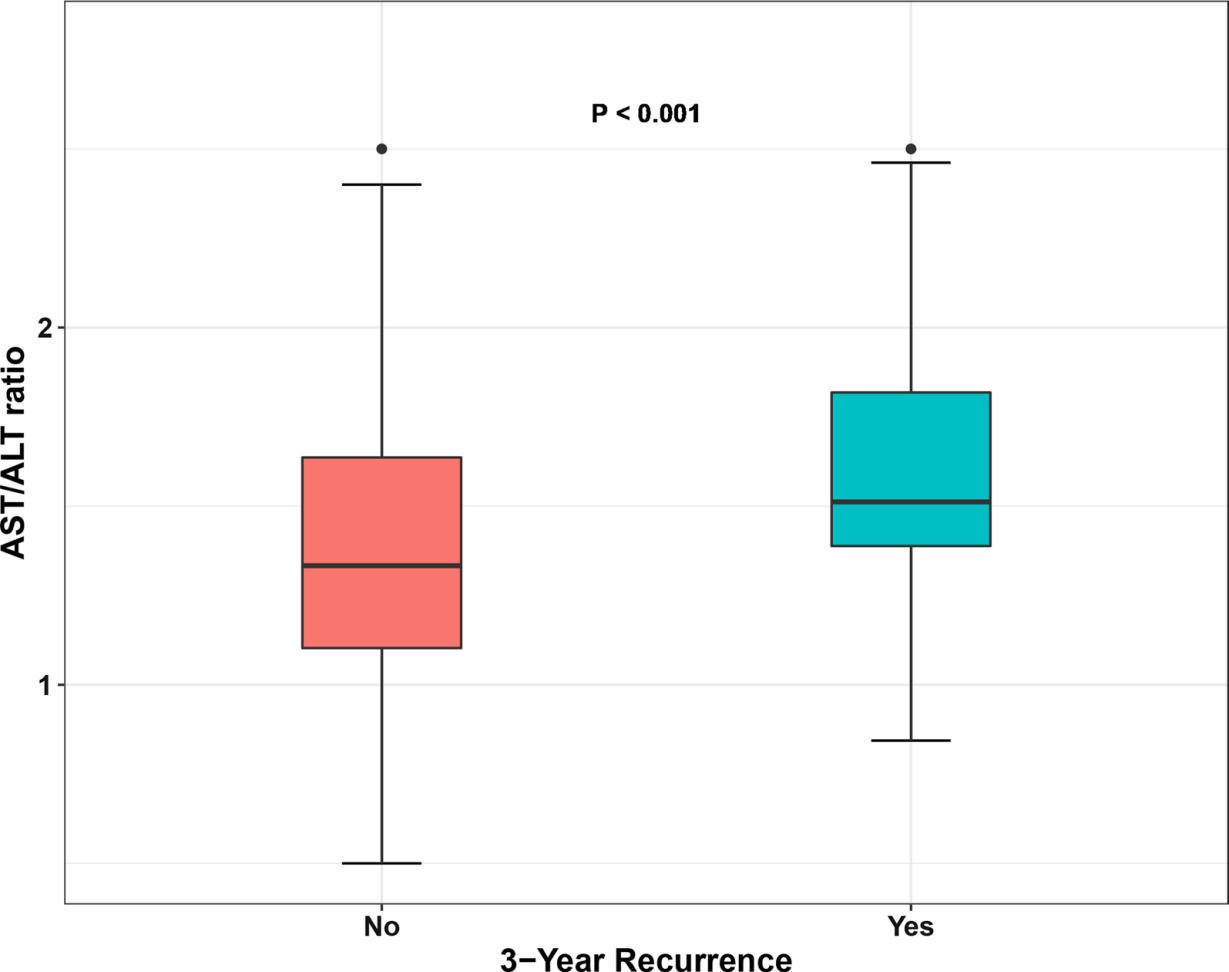
Comparison of the AST/ALT ratio between 3-year recurrence group and 3-year nonrecurrence group

**Fig. 2:**
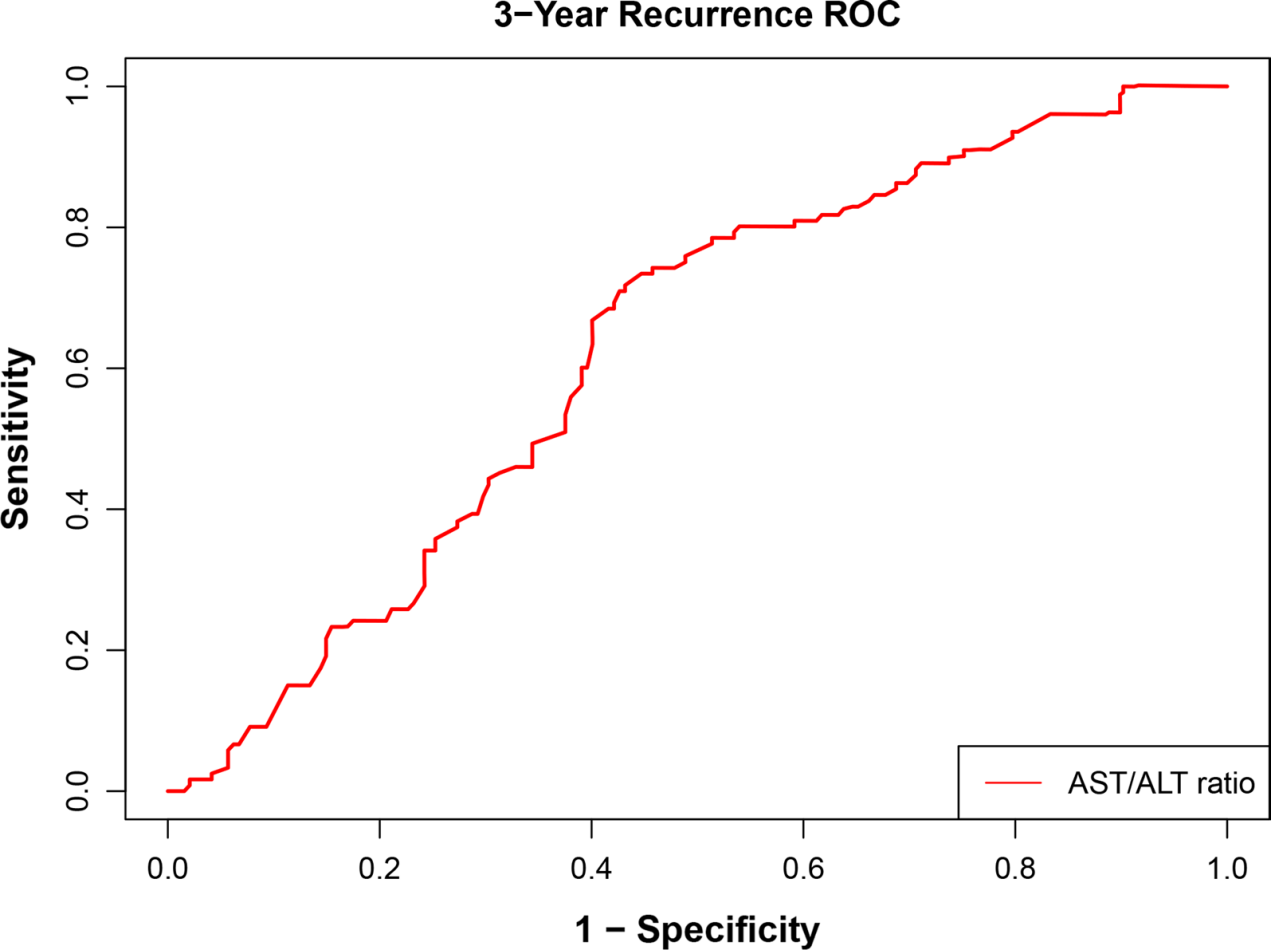
ROC curve analysis for determination of optimal cutoff values of the AST/ALT ratio in 3-year RFS

The median ALT, AST, and the AST/ALT ratio were 16.0 U/L (IQR 12.0–21.0 U/L), 22.0 U/L (IQR 19.0–27.0 U/L), and 1.4 (IQR 1.2–1.8), respectively. Patients who relapsed within 3 years after initial TURBT was more tend to have a high AST/ALT ratio (*P* < 0.001) (Fig. 1). According to the receiver operating characteristic curve for 3-year recurrence-free survival, when there is a maximum value of the Youden index, the optimal cutoff value for the AST/ALT ratio is 1.40 (area under the curve 0.627, sensitivity 0.746, specificity 0.541) (Fig. 2). In the full cohorts, there were 139 (44.3%) patients in the high AST/ALT group and 175 (55.7%) patients in the low AST/ALT group.

### Relationship between preoperative AST/ALT ratio and Clinical and pathological characteristics in the full and PSM cohorts

With regard to the clinical characteristics of the two groups in the full cohorts, there was no statistical difference between the two groups in gender, history of smoking, Re-TURBT, tumor size, tumor number, pathological grade, concomitant carcinoma in situ (CIS), and type of intravesical therapy (all *P* ≥ 0.05). However, the pathological T stage of the high AST/ALT ratio group was worse than those of the low AST/ALT ratio group (*P* < 0.001), and the proportion of those aged > 60 years was also higher (*P* = 0.007) (Table 1). After 1:1 PSM, the cohort included 100 patients, of which 102 (50.0%) were in the high AST/ALT group and 102 (50.0%) in the low AST/ALT group. There were no statistically significant differences in reported baseline covariates in PSM cohorts (Table 1).

**Table1:** Clinical and pathological data of all patients and propensity score-matched cohorts

Variables	Before PSM			After PSM		
	Low AST/ALT ratio (n = 139)	High AST/ALT ratio (n = 175)	*P* value^a^	Low AST/ALT ratio (n = 102)	High AST/ALT ratio (n = 102)	*P* value^b^
*Age, n (%)*			0.007*			0.597
≤ 60 year	59 (42.4)	49 (28.0)		31 (30.4)	35 (34.3)	
> 60 year	80 (57.6)	126 (72.0)		71 (69.6)	67 (65.7)	
*Gender, n (%)*			0.133			0.728
Female	23 (16.5)	41 (23.4)		18 (17.6)	21 (20.6)	
Male	116 (83.5)	134 (76.6)		84 (82.4)	81 (79.4)	
Smoking, n (%)	67 (48.2)	70 (40.0)	0.146	49 (48.0)	52 (51.0)	0.736
*Tumor size, n (%)*			0.117			1.000
≤ 3 cm	115 (82.7)	132 (75.4)		83 (81.4)	83 (81.4)	
> 3 cm	24 (17.3)	43 (24.6)		19 (18.6)	19 (18.6)	
*Tumor number, n (%)*			0.084			0.711
Solitary	74 (53.2)	76 (43.4)		50 (49.0)	53 (52.0)	
Multiple	65 (46.8)	99 (56.6)		52 (51.0)	49 (48.0)	
Re-TURBT, n (%)	37 (26.6)	49 (28.0)	0.785	27 (26.5)	28 (27.5)	1.000
*Pathological stage, n (%)*			< 0.001*			1.000
pTa	94 (67.6)	67 (38.3)		57 (55.9)	58 (56.9)	
pT1	45 (32.4)	108 (61.7)		45 (44.1)	44 (43.1)	
*Pathological grade, n (%)*			0.954			0.291
LG or less	75 (54.0)	95 (54.3)		50 (49.0)	58 (56.9)	
HG	64 (46.0)	80 (45.7)		52 (51.0)	44 (43.1)	
Concomitant carcinoma in situ, n (%)	11 (7.9)	18 (10.3)	0.471	11 (10.8)	9 (8.8)	0.815
*Type of intravesical therapy, n (%)*			0.331			0.076
Early single instillation	17 (12.2)	17 (9.7)		7 (6.9)	16 (15.7)	
Adjuvant chemotherapy or BCG	25 (18.0)	23 (13.1)		19 (18.6)	14 (13.7)	
Combined therapy	97 (69.8)	135 (77.1)		76 (74.5)	72 (70.6)	

PSM, Propensity score-matched; Re-TURBT, restaging transurethral resection of the bladder tumor; LG or less, low grade or papillary urothelial neoplasms of low malignant potential; HG, high grade; BCG, bacillus Calmette-Guerin

*Indicates *P* < 0.05

^a^χ^2^ test or Fisher’s exact test

^b^McNemar test

### Relationship between preoperative AST/ALT ratio and RFS in the PSM cohorts

**Fig. 3:**
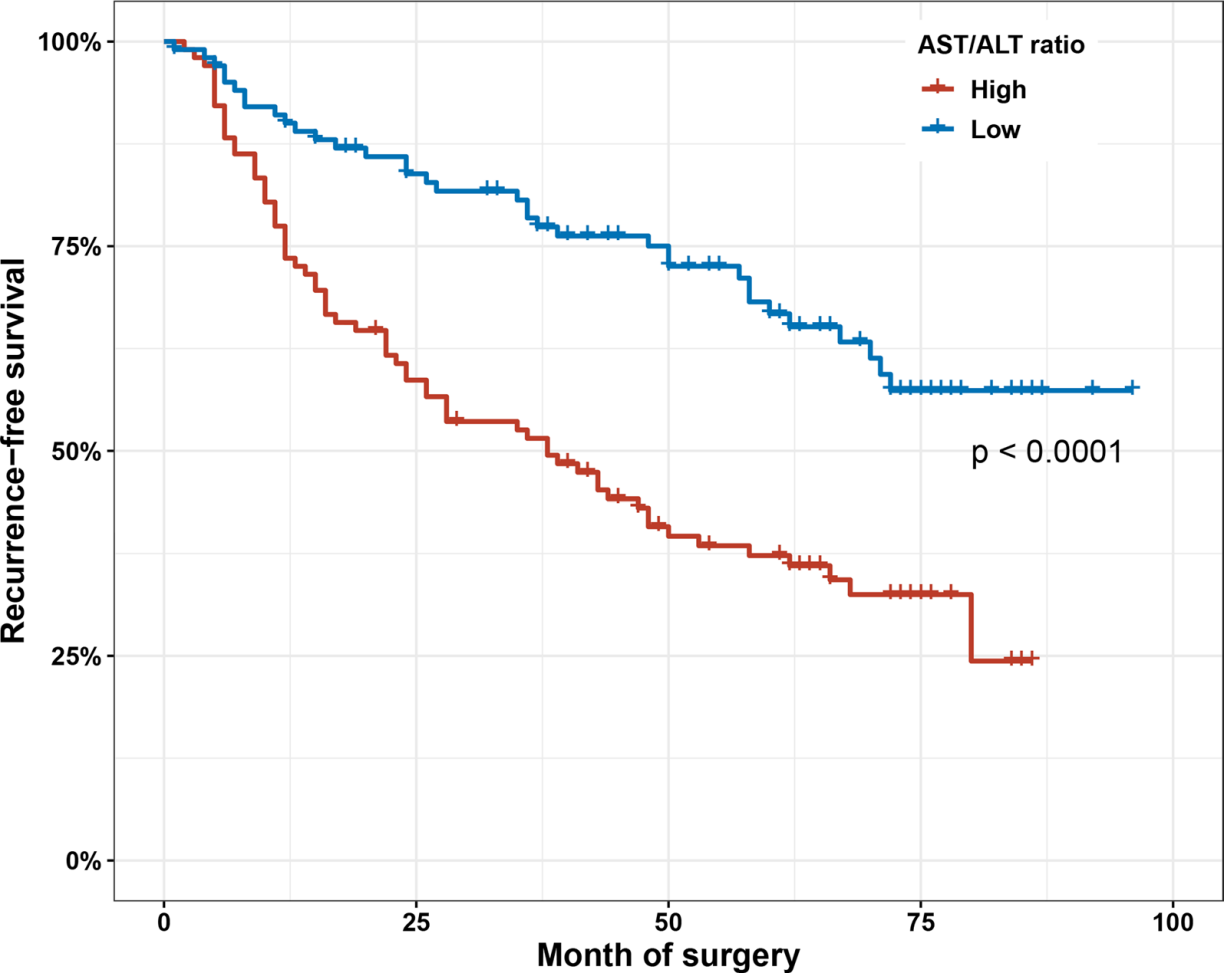
Kaplan–Meier survival curves of RFS according to the AST/ALT ratio in propensity score-matched cohorts

Kaplan–Meier survival analysis was performed to assess the relationship between preoperative AST/ALT ratio and risk of recurrence. The results showed that the low AST/ALT ratio group had significantly better RFS than the high AST/ALT ratio group (*P* < 0.001) (Fig. 3). The univariate Cox analysis of RFS showed that several prognostic factors including age (*P* = 0.010), tumor size (*P* = 0.008), Re-TURBT (*P* = 0.001), pathological stage (*P* = 0.011) and the AST/ALT ratio (*P* < 0.001) were related to the RFS of patients. In multivariate Cox analyses, these related prognostic factors including tumor size (HR 1.879; 95%CI 1.180–2.993; *P* = 0.008), tumor number (HR 1.728; 95%CI 1.140–2.619; *P* = 0.010), Re-TURBT (HR 0.305; 95%CI 0.177–0.526; *P* < 0.001), pathological stage (HR 1.601; 95%CI 1.036–2.474; *P* = 0.034), and the elevated AST/ALT ratio (HR 2.865; 95%CI 1.873–4.381; *P* < 0.001) continued independent association with RFS in multivariate Cox analysis (Table 2).

**Table 2:** Univariate and multivariate cox proportional regression analysis for RFS in propensity score-matched cohorts

Variables	Univariate analysis		Multivariate analysis	
	HR (95%CI)	*P* value	HR (95%CI)	*P* value
*Age, n (%)*				
≤ 60 year	Ref		Ref	
> 60 year	0.596 (0.402–0.884)	0.010*	0.713 (0.476–1.068)	0.101
*Gender, n (%)*				
Female	Ref			
Male	1.039 (0.631–1.712)	0.880		
Smoking, n (%)	1.171 (0.792–1.730)	0.429		
*Tumor size*				
≤ 3 cm	Ref		Ref	
> 3 cm	1.838 (1.168–2.892)	0.008*	1.879 (1.180–2.993)	0.008*
*Tumor number, n (%)*				
Solitary	Ref		Ref	
Multiple	1.428 (0.962–2.119)	0.077	1.728 (1.140–2.619)	0.010*
Re-TURBT, n (%)	0.399 (0.233–0.681)	0.001*	0.305 (0.177–0.526)	< 0.001*
*Pathological stage, n (%)*				
pTa	Ref		Ref	
pT1	1.659 (1.121–2.457)	0.011*	1.601 (1.036–2.474)	0.034*
*Pathological grade, n (%)*				
LG or less	Ref		Ref	
HG	1.301 (0.880–1.923)	0.187	1.336 (0.857–2.081)	0.201
Concomitant carcinoma in situ, n (%)	1.458 (0.814–2.614)	0.205		
*Type of intravesical therapy, n (%)*				
Early single instillation	Ref			
Adjuvant chemotherapy or BCG	1.467 (0.633–3.401)	0.371		
Combined therapy	1.611 (0.777–3.338)	0.200		
*AST/ALT ratio*				
Low	Ref		Ref	
High	2.445 (1.620–3.691)	< 0.001*	2.865 (1.873–4.381)	< 0.001*

RFS, Recurrence-free survival; HR, hazard ratio; CI, confidence interval; Ref, Reference; Re-TURBT, restaging transurethral resection of the bladder tumor; LG or less, low grade or papillary urothelial neoplasms of low malignant potential; HG, high grade; BCG, bacillus Calmette-Guerin

*Indicates *P* < 0.05

### Relationship between preoperative AST/ALT ratio and PFS in the PSM cohorts

**Fig. 4:**
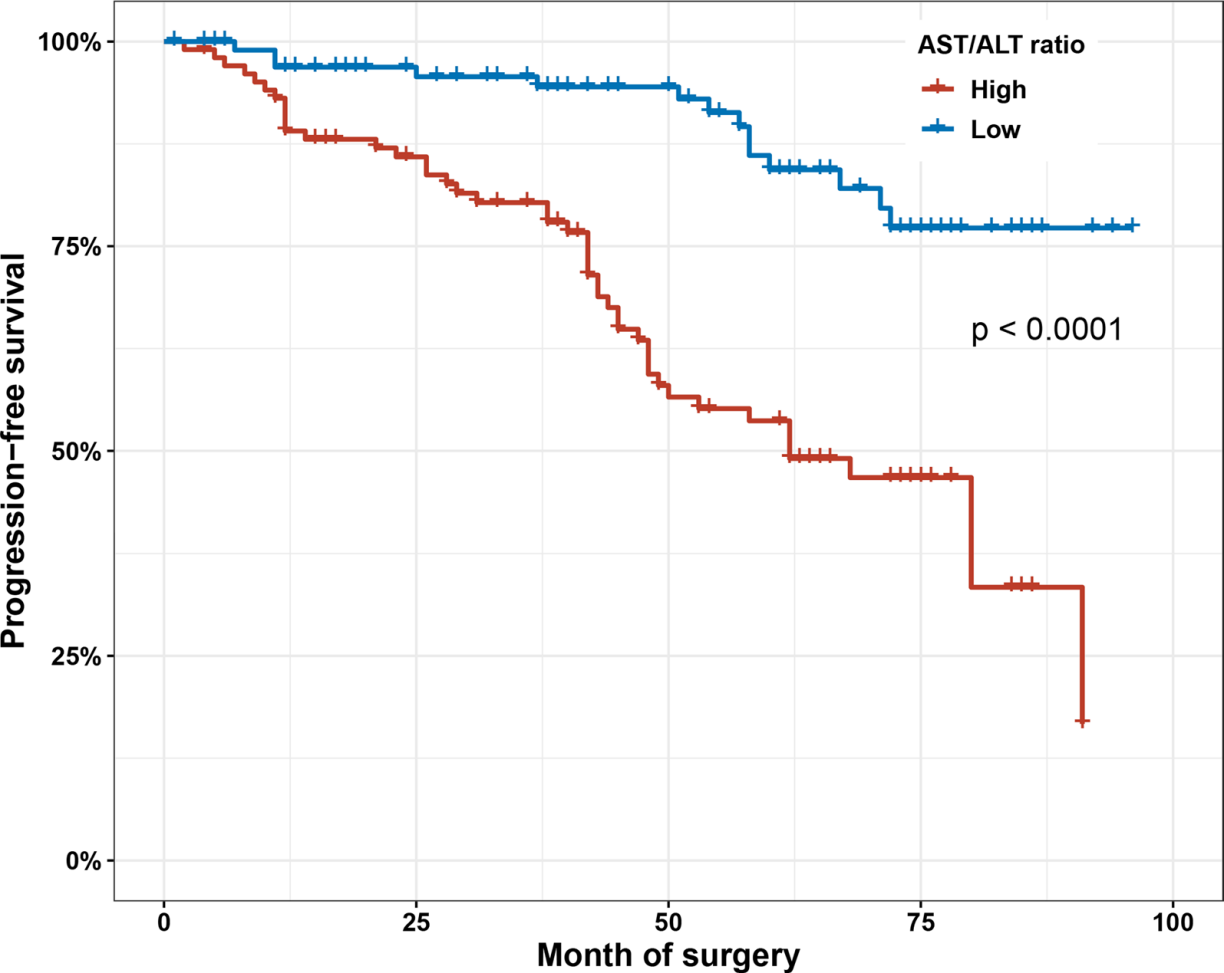
Kaplan–Meier survival curves of PFS according to the AST/ALT ratio in propensity score-matched cohorts

Kaplan–Meier survival analysis demonstrated that the high AST/ALT ratio group tended to have poorer PFS than the low AST/ALT ratio group (*P* < 0.001) (Fig. 4). In univariate Cox proportional hazard model, the prognostic indicators of RFS in primary NMIBC included Re-TURBT (*P* = 0.006), pathological stage (*P* = 0.047), pathological grade (*P* = 0.023), and the AST/ALT ratio (*P* < 0.001). According to Cox multivariate analysis, the independent prognostic indicators of RFS in primary NMIBC involved Re-TURBT (HR 0.301; 95%CI 0.147–0.617; *P* = 0.001), pathological grade (HR 1.893; 95%CI 1.076–3.329; *P* = 0.027), and the AST/ALT ratio (HR 4.771; 95%CI 2.607–8.734; *P* < 0.001) (Table 3).

**Table 3:** Univariate and multivariate cox proportional regression analysis for PFS in propensity score-matched cohorts

Variables	Univariate analysis		Multivariate analysis	
	HR (95%CI)	*P* value	HR (95%CI)	*P* value
*Age, n (%)*				
≤ 60 year	Ref			
> 60 year	0.859 (0.505–1.462)	0.576		
*Gender, n (%)*				
Female	Ref			
Male	0.748 (0.379–1.477)	0.403		
Smoking, n (%)	1.128 (0.680–1.872)	0.641		
*Tumor size*				
≤ 3 cm	Ref			
> 3 cm	1.666 (0.911–3.044)	0.097		
*Tumor number, n (%)*				
Solitary	Ref		Ref	
Multiple	0.982 (0.591–1.632)	0.943	1.877 (0.997–3.532)	0.051
Re-TURBT, n (%)	0.372 (0.183–0.758)	0.006*	0.301 (0.147–0.617)	0.001*
*Pathological stage, n (%)*				
pTa	Ref		Ref	
pT1	1.687 (1.007–2.828)	0.047*	1.534 (0.873–2.694)	0.137
*Pathological grade, n (%)*				
LG or less	Ref		Ref	
HG	1.820 (1.085–3.053)	0.023*	1.893 (1.076–3.329)	0.027*
Concomitant carcinoma in situ, n (%)	1.752 (0.859–3.575)	0.123		
*Type of intravesical therapy, n (%)*				
Early single instillation	Ref			
Adjuvant chemotherapy or BCG	1.061 (0.385–2.920)	0.909		
Combined therapy	1.191 (0.507–2.798)	0.688		
*AST/ALT ratio*				
Low	Ref		Ref	
High	4.165 (2.283–7.599)	< 0.001*	4.771 (2.607–8.734)	< 0.001*

PFS, Progression-free survival; HR, hazard ratio; CI, confidence interval; Ref, Reference; Re-TURBT, restaging transurethral resection of the bladder tumor; LG or less, low grade or papillary urothelial neoplasms of low malignant potential; HG, high grade; BCG, bacillus Calmette-Guerin

*Indicates *P* < 0.05

## Discussion

The serum levels of AST and ALT are biochemical markers widely used in clinical treatment to determine the liver function and to identify liver diseases such as viral hepatitis and alcoholic hepatitis. The AST is widely expressed in several organs, but ALT is s considered more liver-specific and enriched [21]. Thus, pathological conditions leading to a higher proliferative state, tissue damage, and high tumor cell turnover are likely to elevate AST rather than ALT, at least not to the same extent, making the AST/ALT ratio a novel potential biomarker in tumors.

In the present study, we find that the high AST/ALT ratio has independent predicting early disease relapse and progression value in primary NIMBC after PSM. In fact, several recent studies about the AST/ALT ratio all demonstrated that it could be considered as a significant prognostic biomarker in renal cell carcinoma, lung cancer, pancreatic cancer, and colorectal cancer. [11–15]. Lee et al. indicated that the AST/ALT ratio (≥ 1.5) is an independent prognostic factor associated with a worse prognosis of disease progression, overall mortality, and cancer-specific mortality in patients with nonmetastatic renal cell carcinoma [12]. Moreover, Nishikawa et al. also first reported that the AST/ALT ratio was considered as an independent prognostic factor in upper tract urothelial cancer [22]. Similarly, Gorgel et al. [16] Ha et al. [17] and Yuk et al. [18] all concluded that the AST/ALT ratio was an independent prognostic predictor in patients undergoing radical cystectomy with bladder urothelial carcinoma. These findings are consistent with the results of our study. On the contrary, Cho et al. showed that the AST/ALT ratio was not significantly associated with RFS in upper urinary tract urothelial carcinoma [23]. This might be related to different diseases, different pathological states, as well as the selected population.

This study describes the AST/ALT ratio as a novel significant biomarker for predicting recurrence and progression in patients with primary NMIBC. However, the mechanism behind the relationship between NMIBC and AST/ALT ratio remains obscure and requires further research. Several hypotheses have been presented to explain this mechanism. The most famous of these hypotheses is the “Warburg effect.” In tumor cell metabolism, it produces enough adenosine triphosphatase (ATP) by increasing glucose uptake and anaerobic glycolysis to promote the multiplying of cancer cells [24]. Meanwhile, the accelerated glycolysis will promote the secretion of lactate. The study suggested that increased lactate plays an important role in maintaining glycolysis, affecting the ratio of lactate dehydrogenase and nicotinamide adenine dinucleotide (NADH)/NAD^+^ as well as the glucose transporter. [25]. It is known that AST plays an essential role in glycolysis by relocating NADH into the mitochondria by the malate-aspartate shuttle pathway. Consequently, cancer cell metabolism might be connected with the AST/ALT ratio in many glucose-using malignancies. Bladder cancer is considered as a glucose-reliant malignant tumor [26, 27]. Whyard et al. used fluorescence microscopy to investigate the uptake of fluorescent glucose by bladder cancer cells and found that there was a big difference in glucose consumption between normal urothelial and malignant urothelial cells [26]. Therefore, it is highly likely that the AST/ALT ratio is correlated with the prognosis of patients with primary NMIBC after TURBT, but our understanding of the mechanism behind it is so far limited.

Despite the current study showed novel findings, it has some limitations. First, this study was a retrospective analysis. Although all included patients received alike treatment, there were inevitably various inherent biases. Second, the AST/ALT rate was performed only once on the patient before TURBT and no comparative analysis was conducted at the interval of follow-up. Finally, it has the disadvantage of a single-center study and a small sample size. The results of this study need prospective larger studies to further consolidate, but this finding also would help clinicians in clinical decision making.

## Conclusions

This study has shown that the high AST/ALT ratio group tended to have poorer RFS and PFS than the low AST/ALT ratio group. Meanwhile, our results also indicated that the elevated preoperative AST/ALT ratio could be regarded as a significant prognostic biomarker for predicting early disease recurrence and progression of patients with primary NMIBC after TURBT. The findings would further help clinicians in making clinical decisions.

## Data Availability

The datasets used and analyzed during the current study are available from the corresponding author on reasonable request.

## Abbreviations

ALT: Alanine transaminase
AST: Aspartate transaminase
CI: Confidence interval
HR: Hazard ratio
IQR: Interquartile range
MIBC: Muscle invasive bladder cancer
NMIBC: Non-muscle invasive bladder cancer
PFS: Progression-free survival
PSM: Propensity score-matched analysis
Re-TURBT: Restaging transurethral resection of the bladder tumor
RFS: Recurrence-free survival
TURBT: Transurethral resection of bladder tumor
